# Identification of Health Expenditures Determinants: A Model to Manage the Economic Burden of Cardiovascular Disease

**DOI:** 10.3390/ijerph18094652

**Published:** 2021-04-27

**Authors:** Fiorella Pia Salvatore, Alessia Spada, Francesca Fortunato, Demetris Vrontis, Mariantonietta Fiore

**Affiliations:** 1 Department of Economics, University of Foggia, 71121 Foggia, Italy; fiorellapia.salvatore@unifg.it; 2 Sector of Hygiene, Department of Medical and Surgical Sciences, University of Foggia, 71121 Foggia, Italy; francesca.fortunato@unifg.it; 3 School of Business, University of Nicosia, Nicosia 1700, Cyprus; vrontis.d@unic.ac.cy

**Keywords:** health costs, healthcare management, gender medicine, GLM model, GLMM, data oriented

## Abstract

The purpose of this paper is to investigate the determinants influencing the costs of cardiovascular disease in the regional health service in Italy’s Apulia region from 2014 to 2016. Data for patients with acute myocardial infarction (AMI), heart failure (HF), and atrial fibrillation (AF) were collected from the hospital discharge registry. Generalized linear models (GLM), and generalized linear mixed models (GLMM) were used to identify the role of random effects in improving the model performance. The study was based on socio-demographic variables and disease-specific variables (diagnosis-related group, hospitalization type, hospital stay, surgery, and economic burden of the hospital discharge form). Firstly, both models indicated an increase in health costs in 2016, and lower spending values for women (*p* < 0.001) were shown. GLMM indicates a significant increase in health expenditure with increasing age (*p* < 0.001). Day-hospital has the lowest cost, surgery increases the cost, and AMI is the most expensive pathology, contrary to AF (*p* < 0.001). Secondly, AIC and BIC assume the lowest values for the GLMM model, indicating the random effects’ relevance in improving the model performance. This study is the first that considers real data to estimate the economic burden of CVD from the regional health service’s perspective. It appears significant for its ability to provide a large set of estimates of the economic burden of CVD, providing information to managers for health management and planning.

## 1. Introduction

The ongoing evolution of demographic dynamics, and the consequent modification of the population’s health needs, with a growing share of elderly patients and those with chronic diseases, requires health systems to be structurally and organizationally redesigned [1].

Chronic diseases represent the close time horizon of the world countries, given the constantly increasing trend of risk factors [2]. Increasing costs occur when complications occur, thus emphasizing the necessity of secondary preventative approaches in health management [3]; actively preventing complications can control a disease’s impacts on one’s quality of life, decreasing costs, and improving the health system [4]. National health services are currently challenged to research and develop strategies, determinants, and impacts to reduce the predisposition to chronic degenerative diseases and to reduce the burden of the same on public accounts. A careful economic evaluation of the determinants reveals the paramount importance of starting new cost-effective strategies to optimize performance of health expenditure [5].

Around the world, governments, academia, and experts in health economics are faced with increased healthcare costs and are searching for tools to reform and to reduce health expenditures [6,7]. Healthcare costs are increasing more and more across all of the world’s countries and represent a significant burden on each government [8]. The multiple effects of chronic diseases on a country’s productivity and demography appears crucial for defining the performances of a national economy [9,10]. Tracing and investigating the characteristics of chronic diseases appears crucial for identifying the burden of disease, for better managing health services, and for assessing prevention [11,12,13]. However, the determination of economic burden remains the most complex part of the general assessments.

Cardiovascular disease (CVD) is the principal cause of mortality worldwide, which determines high costs of medical care [2,14,15,16,17,18,19]. In order to predict CVD, based on general determinants, Mozos [20] reviewed the main literature on CVD and risk factors linked to shift work (hypertension, cardiac arrhythmia, coronary heart disease, stroke, arterial stiffness, and early arterial aging). Results showed that shift work determines relevant biological, behavioral, physiological, and psychosocial stress, affecting cardiovascular diseases. Koçkaya et al. [21] performed a direct cost analysis of managing comorbidities of CVD with the reimbursement authority’s perspective. Three phases were carried out: (1) a survey questionnaire administrated to five experts in the cardiology field; (2) an expert panel to review the questionnaire filled by all the five experts; and (3) three experts of the panel re-filled the questionnaire as per the daily clinical practice. The selected common comorbidities (CCs) were major bleeding, minor bleeding, stroke, myocardial infarction, and intracranial bleeding: acute cost and chronic maintenance cost were calculated for the different CCs. The results underlined and evidenced the extra economic burden gained from in the comorbidity conditions of CVD (among the latter one, myocardial infarction was found to have the highest burden).

Adepu et al. [22] investigated the correlation between socioeconomic determinants of health (SDH-physical inactivity, income, health insurance status, urban–rural status, air quality) and CVD in Georgia by carrying out a multivariate regression model between 2014 and 2016. Results showed that a lower median household income and yearly average concentrations of fine dust particles (PM 2.5) were the most significant factors explaining CVD mortality, thus calling for an assessment of policies and interventions aimed at improving socio-economic and environmental status. On the other hand, Timóteo et al. [19] highlighted that CVD determines a considerable consumption of healthcare resources and MI (myocardial infarction)-related loss of labor productivity (indirect costs) in Portugal of over 10 million Euros. The analysis involved 299 patients admitted to a single center aged under 66 years and included demographic characteristics, clinical variables, treatment, and outcome as well as employment status and monthly wage. The latter were estimated by collecting data according to gender and age by the Ministry of Labor database and by the Bank of Portugal. A linear regression analysis was performed to identify predictors of absent days from work. Results highlighted that the most significant clinical predictors were age, preceding MI, STEMI (segment-elevation MI), and male gender in STEMI patients. From these results, the authors stressed the importance of cardiac rehabilitative measures in order to promote an earlier return to work.

In line with this result, Rudawska [10] discussed, compared, and analyzed statistics (by taking into account direct, indirect, and immeasurable costs) to highlight how much the burden of chronic diseases affects the economy of European countries. The work focused on national statistics related to the burden of CVD in OECD countries and carried out comparative analysis and descriptive analysis. Direct, indirect, and immeasurable costs were taken into account and the disability-adjusted life year index was also selected to calculate the burden of chronic diseases. The results demonstrate that indirect costs appear higher than direct costs. Finally, Rudawska’s results clearly showed that the economic growth of a country depends on the public burden of diseases.

Several authors from different countries have investigated the determinants affecting health expenditures [5,23,24,25,26,27], healthcare spending, and its services [24,28]. Several cost-of-illness (COI) studies have evaluated the costs of a single disease in numerous countries [29,30,31]. COI represents a set of aggregate costs (derived from surveys and archive sources) of a particular disease to economy/society, which comprises the direct costs of treating the disease (costs for diagnosis, treatment, and management of disease progression and patients’ own costs), as well as indirect costs, such as loss in productivity resulting from time off from employment. A very small number of literature reviews have been devoted to analysis of the determinants influencing CVD costs. However, use of data and a COI model to estimate the economic burden of CVD from the regional health service’s perspective has not been previously studied.

Hospital discharge data are usually used to address issues of public safety, including the identification of disease rates, patient characteristics, hospitalization costs, and outcomes for a specific disease, spreading the development of disease prevention and control programs. Discharge data can be useful to better understand hospitalization patterns for an exact area, to plan for better allocation of resources, to identify services that a population lacks, and to assess the potential impact of hospital changes [32].

The disadvantages of models used in the literature are different. First, they do not consider the intra-patient variance. Second, a perspective disadvantage is identified: In the literature, the studies used a “patient-cost” or “patient-insurance” perspective while in our research, a “regional-system” perspective is used.

This work attempts to fill this gap and aims to define the determinants that affect the costs of CVD in the regional health service of Italy’s Apulia region in the 2014–2016 period by carrying out and then identifying the best model (GLM or GLMM). Therefore, the study supports the investigation field of determinants of health sector expenditure.

The present research is organized as follows: The next section defines the study design, population, and variables used in the study taking into account two models of investigation. Then, the results are presented, and discussions are argued. Finally, the work closes and concludes with limitations and implications for the healthcare sector.

## 2. Materials and Methods

### 2.1. Study Design

This retrospective longitudinal population-based study was conducted using a large regional health administrative database. Data for patients with acute myocardial infarction (AMI), heart failure (HF), and atrial fibrillation (AF) were extracted from electronic medical records included in the hospital discharge registry (HDR). The HDR is held at the regional epidemiological observatory in the Apulia region, in southern Italy, which has a population of about 4 million (6.67% of the Italian population). HDR collects data on discharge diagnoses (one main and up to five secondary diagnoses) and procedures of all admissions to regional health service (RHS) hospitals in the Apulia region, using the International Classification of Diseases, Ninth Edition, Clinical Modification (ICD-9 CM) coding system for diagnoses.

In addition, this registry contains information on ordinary inpatient hospitalizations and day-hospital services delivered to all patients by healthcare organizations managed by the regional service, and healthcare services provided by the independent sector that the RHS commissioned. RHS is funded by the National Health System and ensures ‘unlimited coverage’ to all its residents, who generally should pay only part of the costs of drugs or services (ticket). Patients who have a specific condition, such as a severe chronic disease, are exempted from co-paying.

This study was conducted from the RHS’ perspective using healthcare costs. Unit costs of medical records were applied to estimate direct healthcare costs. Since RHS updates its data every three years, the last available period for carrying out the analysis was 2014–2016. All reference costs for this period were included. The approval of the ethics committee was not requested as all the data processed were treated in an aggregate manner with full respect of privacy.

### 2.2. Study Population

In order to estimate CVD costs and their evolution over time, a unique database matching the records extracted from data sources by using the personal anonymous ID code was created.

Any patient who, during the observational period, was discharged (excluding voluntary and inter-ward discharge) from an Apulian hospital with a diagnosis of AMI, AF, or HF was considered. The first occurrence of a diagnosis code in at least one of the six discharge diagnosis fields was identified as the inclusion criterion. The following groups of ICD-9-CM codes were considered: (1) 410 for AMI; (2) 427.31 and 427.32 for AF; (3) 428 for HF [33]. The description of the ICD-9-CM codes for each disease of interest is shown in Table 1.

Residents that were hospitalized outside the regional territory of competence were excluded from the analysis. Furthermore, according to Italian legislation, the principal diagnosis coding needs to be based on the health status diagnosed at the end of hospitalization. It represents the main cause of some treatments and/or diagnostic tests used and is mainly responsible for one’s use of health resources [34]. The index date was identified as the date of occurrence of the first criterion above.

### 2.3. Study Variables

The hospital discharge registry (HDR) collects data of all hospitalizations and represents a valid tool for clinical-cost assessments of the use of healthcare resources. In the HDR, the diagnosis-related group (DRG) code appears to be the key element of the records of each patient admitted to a care organization. The DRG code refers to a system that classifies all patients discharged from a care organization into homogeneous groups by absorption of committed resources. This code is attributed by a software called “*DRG-grouper*” and was developed to classify all medical records, to define the categories based on the clinical and demographic information collected for each hospitalization through the hospital discharge form, and to identify each subject to whom information is attributed on diagnoses, any surgical interventions and diagnostic procedures, age, and on the hospital-discharge type [35].

In this study, the only dependent variable is the economic burden of the hospital discharge form (hereafter, *hdf_value*). *Hdf_value* is a variable strictly correlated with the DRG. It represents an economic and monetary appraisal (in Euros) of diverse healthcare services involving the patient’s life during the hospitalization, such as practitioner consultations, inpatient stays, emergency department visits, laboratory and imaging tests, etc.

#### Explanatory Variables

Two types of variables were used: socio-demographic (gender, age, citizenship, residential location in the region and the province), and disease-specific variables. Citizenship was assigned according to the ISTAT country code. The list of foreign countries, with the related codes, is constantly updated following changes in the global geopolitical configuration. Each foreign country is identified by the statistical code and the geographical name (source: https://www.istat.it/it/archivio/6747 accessed on 2 March 2021). The sample’s citizenship was identified with the following codes: (1) European citizenship; (2) African citizenship; (3) Asian citizenship; (4) American citizenship; (5) Oceania citizenship; (999) stateless. Mozaffarian et al. [17] divided all CVD patients, identified in the three years observed, into five age classes: (1) under 20; (2) 20–39 years; (3) 40–59 years; (4) 60–79 years; (5) over 80.

Second, the disease-specific variables AMI, AF, and HF were associated with every patient. Each of the variables implies healthcare costs associated with inpatient care. The variables considered were: *DRG, hospitalization-type, hospital stay, surgery* (presence or absence), and *hdf_value*. The healthcare costs from the RHS viewpoint were calculated. Costs were computed using charges that the RHS reimbursed to the healthcare providers.

The economic evaluation of the HDR is given by the monetary value (in Euros) assigned to each DRG. *DRG* is a discrete variable that, as aforementioned, represents the hospital’s “final product” classification tool.

*Hospitalization-type* is a dummy variable for classifying patients based on the type of hospitalization. This variable includes either the ordinary hospitalization option or the day-hospitalization one.

*Hospital stay* is a day-count variable and indicates the patient’s stay in the healthcare organization.

*Surgery* is a dummy variable indicating whether a patient has undergone surgery.

A more detailed explanation of all the variables used in the study can be deepened in Supplementary Materials Table S1. Data on prescription pharmaceutical costs during inpatient stays were not available, so were not included.

### 2.4. Statistical Models and Analysis

**Figure 1:**
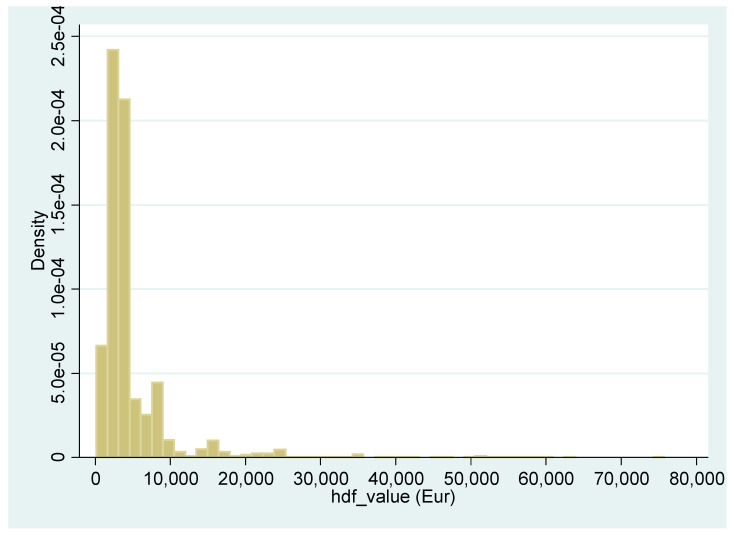
Histogram of *hdf_value*.

Descriptive statistics for each variable were performed. The Kruskal–Wallis test was carried out to determine if there were statistically significant differences between categorical variables on *hdf_value*. In order to analyze the determinants influencing the economic burden of the hospital discharge form, two models were considered: The generalized linear model (GLM) (model 1) and generalized linear mixed model (GLMM) (model 2), being in the presence of heteroscedasticity and random effects [36]. In addition, the distribution of expenses is often skewed to the right because of a moderate number of hospitalizations that involve high expenses (Figure 1), and these models allow use of the link log function.

The GLM (model 1) generalizes a linear model and is composed of two fundamental elements. The first is the link function between the expected value of the result and the linear predictor, and the second one is the variance function, in which the variance is expressed as a function of the mean. To explain the structure of the GLM, the linear model starts from:

(1) E(Y)=μ

where *μ* = *Xβ*. In order to generalize the model indicated in Equation (1), three parts must be considered. The first one is the random part, according to which the components of *Y* have independent Gaussian distributions with *E(Y)* = *μ* and constant variance *σ*^2^. The second part is the systematic component in which the covariates *x*_1_, *x*_2_, … *x_h_* yield a linear predictor *η*, given by:

(2) η=Xβ

Finally, the third part is the link between the random and the systematic component:

(3) μ=η

Given *η_i_* = *g*(*μ_i_*), *g* (.) is the link function (for example, normal, gamma, binomial, Poisson, etc.). The distribution family, based on the modified Park test (*λ*), was chosen. According to Park [37], if the value of the test gives a coefficient *λ* = 0, it indicates that the best choice is the Gaussian family; if *λ* = 1, the Poisson family is the best choice; if *λ* = 2, the gamma family; and if *λ* = 3, the inverse Gaussian family. In this study, the coefficient was 2, thus this sample follows the gamma distribution.

The second model applied was the GLMM (model 2). The GLMM is an extension of the GLM. The GLM includes in the mathematical model only the fixed effects (in this case, age, year, DGR, etc.), estimating their influence on the dependent variable (*hdf_value)*. Instead, the GLMM considers, in addition to the fixed effects, the random effects, i.e., blocks in observational studies or experiments, which are replicated across sites or times [38]. The random effects of the GLMM were the patients, since the variability of costs may depend on the intrinsic characteristics of the patient, which enter the register several times. The general form of the mixed model is:

(4) Y=Xβ+Zu+e

where *Y* is the vector of observations, *X* is the matrix of known constants associated with the fixed effects, *β* is the vector of fixed effects, *Z* is the matrix of known constants associated with the random effects, *u* is the vector of random model effects, and *e* is the vector of random errors [39].

Both model 1 and model 2 were specified as in the gamma family and with link log. The goodness of fit of the models was tested by the Akaike information criterion (AIC) and Bayesian information criterion (BIC); the best model is the one that minimizes these values. All statistical analyses were conducted in Stata 14.0 (Stata Corp LP, College Station, TX, USA). A critical value of *p <* 0.05 was specified a priori as the statistical significance threshold for all analyses.

## 3. Results

### 3.1. Sample Characteristics

In total, 98,829 eligible subjects identified. The sample was aged 75.46 years (1–107) on average, and consisted of 52.8% males and 47.2% females. Most of the patients were Italian (99.1%) and 0.4% were of European nationality. As regards the pathologies investigated, AF was most commonly detected among the Apulian residents (39.5%). Instead, the patients analyzed whom presented two of the three pathologies at the same time were suffering from HF and AF (17.7%), HF and AMI (3.8%), and AF and AMI (1.6%). Only 1.3% of the sample had all three diseases at the same time (Table 2).

### 3.2. Descriptive Statistics

The *hdf_value* varies greatly, showing very high standard deviation values (Table 3).

Regarding gender, *hdf_value* was significantly higher for males (males 4916.15 ± 4969.12 mean ± SD Euros, females 4197.89 ± 4129.42 mean ± SD Euros, Kruskal–Wallis test = 1142.823, *p* < 0.001). As for age, the most expensive age group is the under 20 (5459.21 ± 8460.20 mean ± SD Euros), while the least expensive is the under 39 (4054.95 ± 4247.45 mean ± SD Euros). Overall, the cost differences between the age groups were significant (Kruskal–Wallis test = 357.290, *p* < 0.001). As regards the type of hospitalization, ordinary hospitalization is on average considerably more expensive than day hospitalization (4624.65 ± 4636.66 mean ± SD Euros vs. 2221.47 ± 1405.62 mean ± SD Euros, Kruskal–Wallis test = 1142.823, *p <* 0.001). The presence of surgery also has a large impact on costs (6082.95 ± 6086.81 mean ± SD Euros surgery vs. 3282.49 ± 1992.35 mean ± SD Euros no surgery, Kruskal–Wallis test = 14,691.171, *p* < 0.001). Over the years, there has also been a statistically significant increase in costs (year 2014: 4517.89 ± 4657.98 mean ± SD Euros, year 2016, 4670.34 ± 4636.97 mean ± SD Euros, Kruskal–Wallis test = 173.5551, *p <* 0.001). For patients with a single disease, the most expensive pathology was AMI while the least expensive was AF (AMI: 7775.32 ± 6316.08 mean ± SD Euros, AF: 3724.67 ± 4328.88 mean ± SD Euros, Kruskal–Wallis test = 6846.585, *p* < 0.001).

Among the comorbidities, the largest gap is between HF&AF and AMI&HF (HF&AF: 4975.27 ± 4517.73 mean ± SD Euros, AMI &HF: 7836.86 ± 4313.88 mean ± SD Euros, Kruskal–Wallis test = *p* < 0.001).

**Figure 2:**
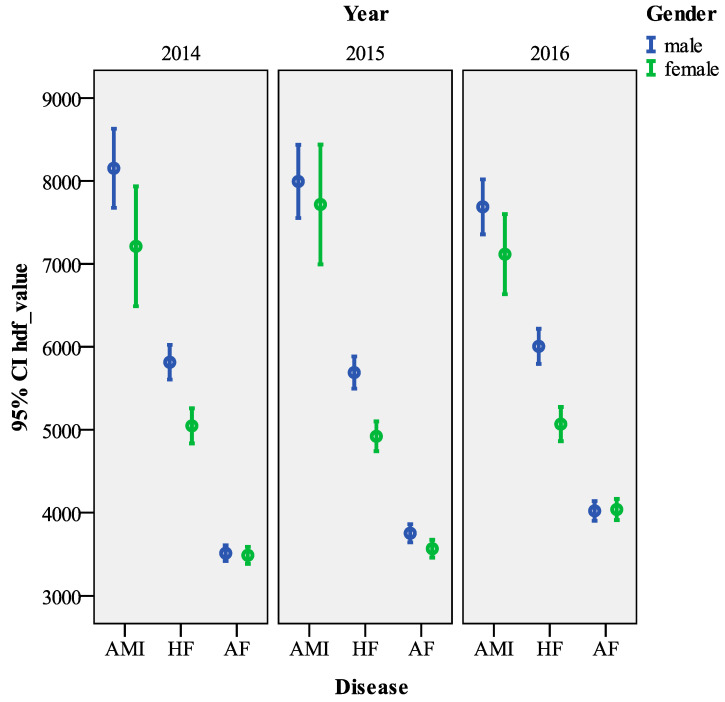
Errorbar chart *hdf_value* by single disease and gender (95% CI).

**Figure 3:**
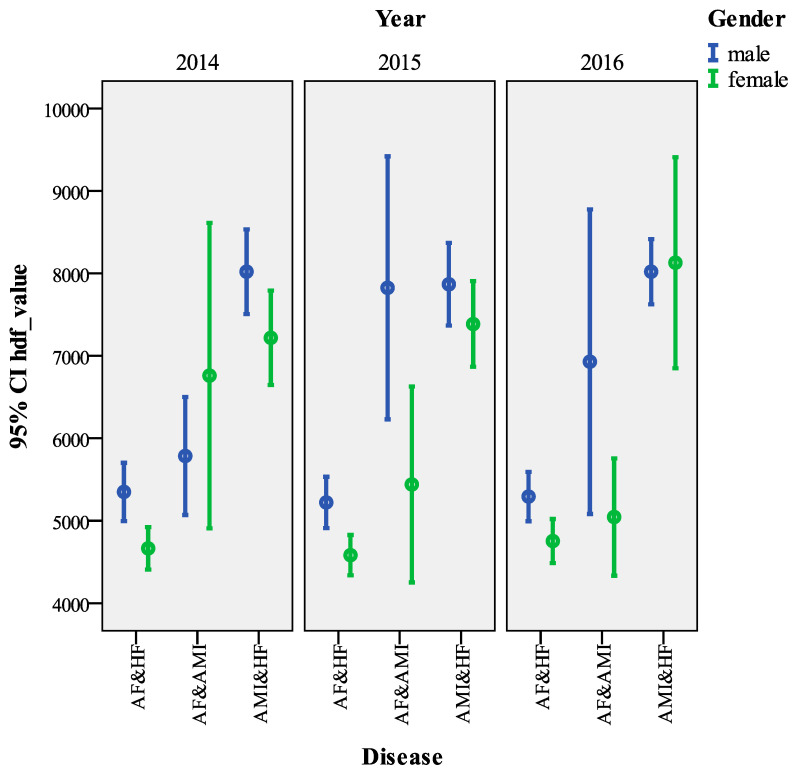
Errorbar chart *hdf_value* by double disease and gender (95% CI).

Finally, Figure 2 (single disease) and Figure 3 (double disease) show the *hdf_value* errorbar charts with 95% confidence intervals, by sex and year, highlighting a gender gap in all years, especially for AF&AMI. Regarding gender, except in 2014, *hdf_value* was higher for males than females.

### 3.3. GLM and GLMM Models

To evaluate the determinants that influence the costs, two models were considered. Table 4 shows the results of the GLM and GLMM models. For each of them, a gamma distribution and log link were performed. Both the GLM and GLMM models showed similar results.

Compared to 2014, the models indicate that in 2016, *hdf_value* recorded an increase (for GLM and GLMM models *p* < 0.001). The spending is on average lower for females (all models *p* < 0.001). GLMM indicates a significant increase in health expenditure with increasing age (*p* < 0.001). Although the category under 20 is the most expensive in terms of care (Table 3), young patients have very little impact on costs in general, being a category that suffers very little from this type of pathology. On the other hand, CVDs greatly increase in frequency in old age, with a greater impact on costs, as evidenced by both models. As expected, due to the nature of the covariates, with the increase of the DRG and hospital stay, there is a significant increase in the *hdf_value* (both models *p* < 0.001). Regarding the hospitalization type, the day-hospital modality results in a lower cost than the ordinary hospitalization modality (both models *p* < 0.001) and surgery increases the cost (both models *p* < 0.001). As for the pathologies, considered individually, the most expensive is AMI, while the cheaper is AF (for both models *p* < 0.001). The two models agree on two important aspects: The absolute values of the coefficients for each determinant are often very similar, and the coefficients are often significant in both models. These results’ concordance indicates robustness, confirming that the included determinants have significant relevance in costing. Comparing the goodness of fit, the GLMM model showed better adequacy (lower values of AIC and BIC). This indicates that the patient has an important role in the variability of the phenomenon, improving the performance of the model.

## 4. Conclusions

The identification of the best model allowed identification of the determinants and their weight influencing the costs of cardiovascular disease by adopting a regional health perspective and using regional data for the period 2014–2016 in the Apulia region (Italy).

As highlighted by the research, although the health administrative databases were not generated with the aim of assessing the economic impact of diseases, they are effective tools for these purposes, as they can provide correct information directly from target populations (not just from samples) observed for related follow-up in a short time period [16]. The research supports the investigation scope of health expenditure determinants from two aspects. First, it represents a surplus compared to the other studies already present in the literature, because this research takes into account determinants of health expenditure not considered in the literature, by inserting them into complex and flexible statistical models, such as the GLMM. They allow for a data structure-oriented model among local geographic territories rather than a standard and inflexible model for investigation, providing information to managers for health planning. Secondly, since most literature has focused on determinants of health expenditures involving national empirical data [27], this study undertook for the first time a comprehensive analysis with real Apulian data using a COI technique.

Specifically, the study focused on three types of CVD—AMI, HF, and AF—that are widespread in the Apulia region. These were selected for different reasons. The AMI pathology in Apulia is classified first for low mortality. This information is confirmed by the data presented here, as the lowest number of patients with AMI disease was identified. This excellent performance, given by the low mortality indicator, is closely related to the efficiency of the service provided by the emergency medicine-urgency departments in the Apulia region. It, therefore, expresses the presence of a regional model of clinical efficacy and management efficiency.

As for HF, the increase in hospitalizations for heart failure has been confirmed by other studies [40]. Data shows that the most worrying problem for health management is the economic and organizational impact on the national health system that this heart disease causes. For this reason, in order to reduce the high rate of early hospitalization and its economic burden on the regional health service, the organizational forecasting of management paths for HF patients is a necessary activity.

The third heart disease object of this study, AF, is a disease destined to increase dramatically over time, reflecting Italy’s demographic trend. From the EUROSTAT data, it can be deduced that, in the elderly population of the 28 European Union countries, the prevalent cases in 2016 were 7.6 million, and they will increase exponentially, reaching 14.4 million in 2060 [15]. These analyses were confirmed in the present study, since the majority of the patients analyzed were affected by AF (39.5% of the sample).

GLM and GLMM models were applied to achieve the main objective of the study, which was to evaluate the determinants that influence the costs. The main findings are provided as follows.

All statistical models found agreement in analyzing which determinants caused an increase and decrease in the *hdf_value*. The models confirmed that in 2016, *hdf_value* increased, and, as the hospitalization stay increases, the *hdf_value* increases. In addition, all models also confirmed some determinants that led to a decrease in the *hdf_value*: female patients as well as the cost of hospitalization in day-hospital generated cost savings. As for the analysis by disease, HF and AMI caused greater expense than AF, which is the cheapest. This result suggests that much should be done, especially in the prevention of AMI and HF diseases. Prevention campaigns that suggest healthier lifestyles and more accurate doctor–patient relationships in the pre-hospital setting could guarantee a lower economic burden on public spending centers. Furthermore, the models confirmed that variable surgery has the greatest impact on the increase of the *hdf_value* (GLM: 0.3370466, GLMM: 0.2874097, *p* < 0.001), compared to the methods of carrying out day-hospital-care, which allows the greatest savings (GLM: −0.5666091, GLMM: −0.6481928, *p* < 0.001). Day hospitalization should also be encouraged compared to ordinary hospitalization when surgery is not necessary. Indeed, increasing the number of day hospitals for each patient, at the expense of longer hospitalizations, would allow better prevention without leading to more serious illness situations.

The comparison of the two models highlighted that the best model is the GLMM, as the Akaike and Bayesan information criteria have the lowest values (Table 4). Since the GLMM model is the only one that considers the random effect and this is the patient, it means that the economic burden sustained by the region is linked to the patient’s access to the regional health service. This is because each patient’s identity is different from the other. Moreover, by investigating the results of descriptive statistics and statistical models, it can be argued that female patients generate cost savings. Although Mosca et al. [41] state that women experience greater morbidity and mortality than men after being diagnosed with cardiovascular disease, they are able to generate savings for the regional health service. In other words, decades of research shows that early identification in women, and particular attention to risk factors, such as high blood pressure, high cholesterol levels, obesity, family history of cardiovascular disease, and diabetes, can substantially reduce disease economic burden and deaths. Lifestyle changes, smoking cessation, and the right use of drugs are proven mainstays of women’s habits that allow greater cost-effectiveness for the regional health service [41,42]. In addition, this result confirms the validity of the recent approach to gender medicine, since significant gender disparities are prevalent in presentation, management, and outcomes of adults with cardiovascular diseases [43,44].

The present study has some potential limitations. First, the comparison with other studies in the literature regarding the self-explanatory results of this study poses limitations due to the originality of our study. Since, to our knowledge, no other study in the literature has used models in the same way, comparing our results with those obtained in other studies has limitations. Furthermore, the level of reimbursement given to health organizations is established first by the national health system, and, secondly, by the loans that the national health system has provided to the individual regions. This practice can be a limitation as it is another way in which overall costs are assessed. Third, some healthcare costs were probably underestimated, due to the absence of the following information in the administrative databases: costs related to cardiovascular risk factors (hypertension, diabetes, smoking, drug abuse, atrial fibrillation, family history, and previous personal history of CVD); costs related to outpatient consultations, laboratory tests, and imaging and diagnostic tests; costs related to prescribed medications; costs related to the use of intensive care units; costs related to the performance of physiotherapy sessions; and post-discharge costs. Finally, the costs of visits to general practitioners (GPs) were not considered. Almost all residents in Italy are enrolled with a GP, from whom they receive primary care in case of need. GPs are paid on a capitation basis, regardless of the actual care provided to their patients; therefore, it is not possible to quantify the costs attributable to visits for cardiovascular reasons. As future research, it could be useful to integrate administrative databases with new ones in which other types of data, i.e., costs related to inpatient and outpatient consultations, medications, and care treatments, are collected and registered, in order to generate more complete data sources for assessment of the economic burden of diseases.

In conclusion, the findings of this study are of interest to the public healthcare sector, as economic evaluation is one of the most important components of studies focused on public health management. Public organizations of the health system fulfill a crucial role for the provision of public services to citizens [45]. The “public service” concept includes all goods and services that the public administration recognizes as being of social utility and therefore ensures their production, distribution, and supply to guarantee free use to the community [46]. Although public services are very different from each other in technical characteristics and delivery methods, they together respond to a need considered public [47]. Ensuring the well-being of citizens, such as supporting campaigns for cardiovascular risk prevention, is one of the fundamental objectives of the public administration [48]. These organizations, by supporting important prevention campaigns, play a fundamental role in guaranteeing huge savings for the public health system [49]. The statistical models analyzed represent a valid decision-making tool both to assess the value-related determinants influencing diseases and to improve the economic management of the health system as a whole.

## Figures and Tables

**Table 1:** ICD-9-CM diagnosis code and description of AMI, AF, and HF diseases.

Diagnosis	Description
Acute myocardial infarction	410.0 Anterolateral wall
	410.1 Other anterior wall
	410.2 Inferolateral wall
	410.3 Inferoposterior wall
	410.4 Other inferior wall
	410.5 Other lateral wall
	410.6 True posterior wall infarction
	410.7 Subendocardial infarction
	410.8 Other specified sites
	410.9 Unspecified site
Atrial fibrillation and flutter	427.31 Atrial fibrillation
	427.32 Atrial flutter
Heart failure	428.0 Congestive heart failure. unspecified
	428.1 Left heart failure
	428.2 Systolic heart failure
	428.20 Unspecified
	428.21 Acute
	428.22 Chronic
	428.23 Acute on chronic
	428.3 Diastolic heart failure
	428.30 Unspecified
	428.31 Acute
	428.32 Chronic
	428.33 Acute on chronic
	428.4 Combined systolic and diastolic heart failure
	428.40 Unspecified
	428.41 Acute
	428.42 Chronic
	428.43 Acute on chronic
	428.9 Heart failure unspecified

**Table 2:** Patient characteristics.

**Patients’ Sample**	***n* (%) or Mean ± SD**
	98,829 (100)
**Panel A—Socio-demographic characteristics**	
Gender	
Male	52,184 (52.8)
Female	46,645 (47.2)
Age (years)	
Mean ± SD	75.46 ± 12.37
Range	1–107
Marital status	
Married	53,866 (54.5)
Divorced/separate	9561 (9.6)
Widowed	16,418 (16.6)
Single	5409 (5.5)
Undeclared	13,575 (13.7)
Nationality	
Italy	97,941 (99.1)
Europe	402 (0.4)
Asia	50 (0.05)
Africa	82 (0.08)
America	18 (0.01)
Oceania	18 (0.01)
Stateless	333 (0.3)
Times the patient is detected in the register	
Mean ± SD	1.59 ± 1.16
Range	1−25
DRG (Euros)	
Mean ± SD	218.85± 174.17
Range	1−579
Hospital stay (day)	
Mean ± SD	9.50 ± 11.56
Range	1–361
**Panel B—Pathologies characteristics**	
Patients with a single disease	
HF	23,449 (23.7)
AF	39,086 (39.5)
AMI	12,239 (12.4)
Patients with two pathologies	
HF&AF	17,484 (17.7)
HF&AMI	3738 (3.8)
AF&AMI	1536 (1.6)
Patients with all three diseases	
HF&AF&AMI	1297 (1.3)

**Table 3:** Summary characteristics of *hdf_value* by each categorical factor.

Categorical Variables		*N*	*hdf_value* (Mean ± SD, Euros)	Kruskal–Wallis Test
Gender	Male	83,395	4916.15 ± 4969.12	1142.823 ***
	Female	73,677	4197.89± 4129.42	
Age	<20	165	5459.21 ± 8460.20	357.290 ***
	<39	1129	4054.95 ± 4247.45	
	<59	14,444	5261.53 ± 5482.95	
	<79	72,023	4893.93 ± 5213.21	
	Over 80	69,311	4116.49 ± 3569.05	
Hospitalization type	Ordinary hospitalization	154,104	4624.65 ± 4636.66	2676.288 ***
	Day hospitalization	2968	2221.47 ± 1405.62	
Surgery	absence	8434	3282.49 ± 1992.35	14,691.171 ***
	presence	72,732	6082.95 ± 6086.81	
Year	2014	52,603	4517.89 ± 4657.98	173.555 ***
	2015	53,237	4670.34 ± 4636.97	
	2016	51,232	4670.34 ± 4636.97	
Single disease	HF	14,602	5490.92 ± 5185.076	6846.585 ***
	AF	35,494	3724.67 ± 4328.88	
	AMI	3694	7775.32 ± 6316.08	
Double disease	AF&HF	5591	4975.27 ± 4517.73	1528.284 ***
	AF&AMI	330	6407.50 ± 85,488.35	
	AMI&HF	1284	7836.86 ± 4313.88	
Triple disease	HF&AMI&AF	140	7441.93 ± 5389.08	

*** = *p* < 0.001.

**Table 4:** Results of the generalized linear model with gamma distribution and log link.

*hdf_value* (Euros)	Model 1 (GLM)	Model 2 (GLMM)
2014 (Base)	-	-
2015	−0.0004	0.0037
2016	0.0255 ***	0.0238 ***
Male (Base)	-	-
Female	−0.04683 ***	−0.04635 ***
**Age**	0.00039	0.0017 ***
Ordinary hospitalization (Base)	-	-
Day hospitalization	−0.56661 ***	−0.6482 ***
**Drg (Euros)**	0.0014 ***	0.0015 ***
**Hospital stay**	0.0181	0.01616 ***
Absence (Base)	-	-
Presence	0.3370466 ***	0.2874 ***
**HF**		
Absence (Base)	-	-
Presence	0.1392167 ***	0.1672 ***
**AMI**		
Absence (Base)	-	-
Presence	0.1773	0.2202 ***
**AF**		
Absence (Base)	-	-
Presence	−0.05545 ***	−0.0603
_Cons	7.6349 ***	7.5031 ***
**Patient Var**	-	0.0649288
**AIC**	**2,840,619**	**2,833,922**
**BIC**	**2,840,748**	**2,834,062**

*** = *p* < 0.001.

## Supplementary Materials

The following are available online at https://www.mdpi.com/article/10.3390/ijerph18094652/s1, Table S1. Variables deriving from the regional health information system.
