# Supplementary material for: Identification of Health Expenditures Determinants: A Model to Manage the Economic Burden of Cardiovascular Disease
Source: Int J Environ Res Public Health. 2021 Apr 27;18(9):4652. doi: 10.3390/ijerph18094652 (PMC8124329; doi:10.3390/ijerph18094652)
Supplement: Supplementary file 1 [file ijerph-18-04652-s001.zip › ijerph-1166279-supplementary.pdf]

Table S1. Variables deriving from the regional health information system.

NAME	TYPE	DESCRIPTION / OPTIONS
Hdf_value	Dependent variable	economic burden of the hospital discharge form
Gender	Dummy variable	Male<br>Female
Age	Discrete variable	-
Marital status	Categorical variable	Married<br>Divorced/separate<br>Widowed<br>Single<br>Undeclared
Nationality	Categorical variable	Italy<br>Europe<br>Asia<br>Africa<br>America<br>Oceania<br>Stateless
DRG	Discrete variable	It is a system that classifies all patients discharged from a hospital (ordinary or day hospitalization) into homogeneous groups by absorption of committed resources (iso-resources). This aspect makes it possible to economically quantify the absorption of resources and therefore to remunerate each episode of hospitalization. One of the aims of the DRG system is to control and contain healthcare costs.
Hospitalization-type	Dummy variable	Hospitalization is a form of care assistance, guaranteed by the National Health Service, which allows the patient to take advantage of hospital care within a day (day hospitalization) or several days (ordinary hospitalization) for carrying out diagnostic tests, specialist visits, and therapies. The stay in hospital is limited to the time strictly necessary and, once the treatment is completed, the patient returns to his home having the possibility to continue daily activities as much as possible.
Hospital stay	Day-count variable	It is a counter variable that allows to understand how long the patient was hospitalized at the health facility.
Surgery	Dummy variable	If the patient was undergone to surgery during hospitalization, the variable takes the value 1. Vice versa, the value will be 0.
